# GLIS3 regulates transcription of thyroid hormone biosynthetic genes in coordination with other thyroid transcription factors

**DOI:** 10.1186/s13578-023-00979-8

**Published:** 2023-02-15

**Authors:** Hong Soon Kang, Sara A. Grimm, Raja Jothi, Pilar Santisteban, Anton M. Jetten

**Affiliations:** 1 Immunity, Inflammation and Disease Laboratory, National Institute of Environmental Health Sciences, National Institutes of Health, Research Triangle Park, Durham, NC 27709 USA; 2 Integrative Bioinformatics, National Institute of Environmental Health Sciences, National Institutes of Health, Research Triangle Park, Durham, NC 27709 USA; 3 Epigenetics & Stem Cell Biology Laboratory, National Institute of Environmental Health Sciences, National Institutes of Health, Research Triangle Park, Durham, NC 27709 USA; 4 Instituto de Investigaciones Biomédicas “Alberto Sols”, Consejo Superior de Investigaciones Científicas (CSIC), Universidad Autónoma de Madrid (UAM), Madrid, Spain

**Keywords:** GLIS3, NKX2.1, PAX8, FOXE1, Gene transcription, Thyroid follicular cells, PCCl3, Thyroid hormone biosynthesis, TSH, NIS

## Abstract

**Background:**

Loss of the transcription factor GLI-Similar 3 (*GLIS3*) function causes congenital hypothyroidism (CH) in both humans and mice due to decreased expression of several thyroid hormone (TH) biosynthetic genes in thyroid follicular cells. Whether and to what extent, GLIS3 regulates thyroid gene transcription in coordination with other thyroid transcriptional factors (TFs), such as PAX8, NKX2.1 and FOXE1, is poorly understood.

**Methods:**

PAX8, NKX2.1, and FOXE1 ChIP-Seq analysis with mouse thyroid glands and rat thyrocyte PCCl3 cells was performed and compared to that of GLIS3 to analyze the co-regulation of gene transcription in thyroid follicular cells by these TFs.

**Results:**

Analysis of the PAX8, NKX2.1, and FOXE1 cistromes identified extensive overlaps between these TF binding loci and those of GLIS3 indicating that GLIS3 shares many of the same regulatory regions with PAX8, NKX2.1, and FOXE1, particularly in genes associated with TH biosynthesis, induced by thyroid stimulating hormone (TSH), and suppressed in *Glis3*KO thyroid glands, including *Slc5a5* (*Nis*), *Slc26a4*, *Cdh16*, and *Adm2*. ChIP-QPCR analysis showed that loss of GLIS3 did not significantly affect PAX8 or NKX2.1 binding and did not cause major alterations in H3K4me3 and H3K27me3 epigenetic signals.

**Conclusions:**

Our study indicates that GLIS3 regulates transcription of TH biosynthetic and TSH-inducible genes in thyroid follicular cells in coordination with PAX8, NKX2.1, and FOXE1 by binding within the same regulatory hub. GLIS3 does not cause major changes in chromatin structure at these common regulatory regions. GLIS3 may induce transcriptional activation by enhancing the interaction of these regulatory regions with other enhancers and/or RNA Polymerase II (Pol II) complexes.

**Supplementary Information:**

The online version contains supplementary material available at 10.1186/s13578-023-00979-8.

## Introduction

Thyroid hormone (TH) biosynthesis in thyroid follicular cells is regulated by thyroid stimulating hormone (TSH) released by the pituitary [1, 2]. Interaction of TSH with the TSH receptor (TSHR), a G-protein-coupled receptor, causes activation of several protein kinase pathways that subsequently lead to increased transcription of several genes critical for TH biosynthesis, including the sodium–iodide symporter (*NIS*; *SLC5A5*), dual oxidase 2 (*DUOX2*), thyroglobulin (*TG*), thyroid peroxidase (*TPO*), and pendrin (*PDS*; *SLC26A4*) [3, 4]. Impairments in TH biosynthesis lead to thyroid dyshormonogenesis, one type of congenital hypothyroidism (CH) [5–9].

Paired box 8 (*PAX8*), NK2 homeobox 1 (*NKX2.1* or *TTF1*), forkhead box E1 (*FOXE1* or *TTF2*), and hematopoietically expressed homeobox (*HHEX*) are among the transcription factors (TFs) that have been implicated in the regulation of several TH biosynthetic genes as well as embryonic thyroid gland development [3, 4, 6, 10–13]. Mutations in these genes are causally linked to defects in thyroid gland development (thyroid dysgenesis), the major cause of CH [3, 4, 7, 10, 14–16].

Recently, we identified the Krüppel-like zinc finger TF, GLI-Similar 3 (GLIS3), as an additional critical regulator of TH biosynthesis [17–19]. Loss of GLIS3 function in humans and mice causes a syndrome characterized by neonatal diabetes and CH [18–26]. In Zebrafish, *glis3* was found to be critical for early thyroid development [27]. Single nucleotide polymorphisms (SNPs) in human *GLIS3* have been associated with increased risk of CH and thyroid dysfunction [28–36].

GLIS3 directly regulates the transcription of several TH biosynthesis-related genes, including *Slc5a5*, *Slc26a4*, and *Duoxa2* [18, 19]*.* Loss of GLIS3 function greatly reduces the expression of TH biosynthesis-related genes, thereby providing a causal mechanism for the development of CH in *Glis3*KO mice and likely in humans as well [19]. Comprehensive hypergeometric optimization of motif enrichment (HOMER) analysis of our GLIS3 ChIP-Seq data revealed that binding sites for members of the PAX, NKX, and FOX TF families regularly localize near GLIS3 binding loci. We hypothesized that GLIS3 regulates thyroid gene transcription in coordination with TFs with well-established regulatory functions in the thyroid gland, such as PAX8, NKX2.1, and FOXE1. To obtain further support for this hypothesis and to analyze the extent by which GLIS3 co-regulates transcription with these thyroid TFs, we performed PAX8, NKX2.1, and FOXE1 ChIP-Seq analyses in the mouse thyroid gland and rat thyrocyte PCCl3 cells. These analyses indicated that GLIS3 co-regulates the transcription of several genes critical for TH biosynthesis and/or induced by TSH, with these thyroid TFs by binding within the same regulatory hub in these genes. We further show that loss of GLIS3 does not cause major changes in the binding of PAX8 or NKX2.1 or the open chromatin structure at these common regulatory regions. GLIS3 may induce transcriptional activation by enhancing the interaction of regulatory regions with RNA Polymerase II (Pol II) complexes.

## Methods

### Mice

*Glis3*-EGFP mice (C57BL/6-Glis3<tm3(Glis3-EGFP)Amj>) expressing a GLIS3-EGFP fusion protein and *Glis3*-deficient (*Glis3*KO) mice (B6.Glis3<tm3(mCherry)AmJ>) were described previously [19, 37, 38]. Mice were routinely fed a NIH-31 normal diet (ND; Harlan, Madison, WI). For ChIP-Seq, 8–10 weeks-old *Glis3*-EGFP mice were fed a low-iodine diet (LID; TD.95125 diet, Harlan) for 2 weeks before thyroid glands were collected for analysis. All animal studies followed guidelines outlined by the NIH Guide for the Care and Use of Laboratory Animals and protocols were approved by the Institutional Animal Care and Use Committee at the NIEHS.

### Cell culture and Western blot analysis

Rat thyrocyte PCCl3 cells (ATCC CRL-1468) were cultured in Coon’s/F12 as described previously [19]. PCCl3-pIND20-Glis3 cells, expressing a doxycycline (Dox)-inducible GLIS3 tagged with Flag and HA at N- and C-terminus, respectively, were generated via pIND20-Flag-Glis3-HA lentivirus infection and subsequent puromycin selection as described previously [39].

### ChIP-Seq

ChIP-Seq was carried out as described previously [19, 39]. Briefly, thyroid glands from *Glis3*-EGFP mice fed a LID for 2 weeks, were isolated and homogenized in PBS using a Tekmar Tissumizer Homogenizer (Tekmar Company, Cincinnati, OH). Homogenate was cross-linked in 1% formaldehyde for 10 min and the reaction subsequently quenched by the addition of 125 mM glycine for 10 min. In the case of PCCl3-pIND20-Glis3-HA, cells were treated for 24 h with 100 ng/ml Dox and then collected and processed for cross-linking and quenching as described above for the thyroid gland. The cross-linked tissues or cells were washed two times with PBS, resuspended in lysis buffer A for 10 min, pelleted, and resuspended in lysis buffer B for 10 min. Samples were subsequently sheared in lysis buffer C for 40 min using an S220 focused-ultrasonicator (Covaris, Woburn, MA). After centrifugation, the cleared chromatin supernatant was incubated with a PAX8 (NBP1-32440, Novus Biologicals) or NKX2.1 (ab76013, Abcam) antibody for tissue ChIP, and HA (#3724, Cell Signaling), NKX2.1 or FOXE1 (PA02000, Biopat) antibody for PCCl3 ChIP. For ChIP-Seq for histone marks, thyroid gland from 4-week-old WT and *Glis3*KO mice fed ND and LID for 6 days were collected and processed as above. Then chromatin supernatant was incubated with H3K4me3 (ab8580, Abcam) and H3K27me3 (ab6002, Abcam) antibodies. After an overnight incubation at 4 °C, washed Dynabeads Protein G (ThermoFisher Scientific) were added and the mixture rotated for 3 h at 4 °C. After subsequent washes, ChIPed-DNA was eluted, reverse cross-linked, incubated with proteinase, and DNA fragments purified using a PCR purification kit (Qiagen). Libraries were synthesized using a NEXTflex Rapid DNA-Seq kit (PerkinElmer, Austin, TX). Sequencing was performed with NovaSeq 6000, NextSeq 500 or MiSeq (Illumina, San Diego, CA).

### ChIP-seq data analysis

Raw sequence reads were filtered to remove any entries with a mean base quality score < 20. Adapters were removed by Cutadapt v1.12 and single-end reads mapped against the mm10 or rn6 reference assembly via Bowtie v1.2, with only uniquely mapped hits accepted [40, 41]. Duplicate mapped reads were removed via MarkDuplicates.jar (using flag REMOVE_DUPLICATES = TRUE) from the Picard tool suite v1.110. Initial peak calls for TFs from mouse thyroid samples were made by HOMER (v4.10.3) with parameters “-style factor -fdr 0.00001 -F 8”, comparing each ChIP sample against the respective input sample. Initial peak calls for TFs from PCCl3 samples were made by HOMER (v4.10.3) with parameters “-style factor -fdr 0.001 -F 4”, comparing each ChIP sample against the respective input sample. For all TFs, peaks were re-sized to 300 bp centered on the called peak midpoints prior to downstream analysis. Enriched motifs were identified by HOMER ‘findMotifsGenome’ at “-size given” and all other parameters default. For the purposes of the genomic context of peak summaries, TSS proximal is defined as the region from − 1 kb to the annotated transcriptional start site (TSS), Upstream as the region from − 5 kb to − 1 kb relative to TSS, Genebody as the region from TSS to transcription end site (TES), and Intergenic as all other genomic locations. To establish a collapsed set of regions for direct comparison of ChIP-seq signal from multiple TFs, a combined peak set was defined via BEDTools (v2.29.2) merge function with parameter “-d 50”. Each collapsed peak was then scored as positive or negative for each TF according to the probability that the overlapping fragment count would be observed at random (cutoff set at 1e−6).

Peak calls for histone marks were made by HOMER (v4.10.3) with parameters “-region -size 500 -minDist 1000 -L 0” for H3K4me3 and “-region -size 1000 -minDist 2500 -L 0” for H3K27me3. For each histone mark, the called peaks were collapsed to a single unified peak set defined as the union of peak calls from individual samples. Tukey box-and-whisker plots of histone modification ChIP-seq signal were generated with R package ggplot2 (v3.3.2), in which the box indicates the 25th through 75th percentile and the whiskers denote values up to 1.5 * inter-quartile range from the 25th or 75th percentile. Counts of mapped ChIP-seq reads (extended to the estimated fragment length of 150 bp) overlapping ± 1 kb flanks relative to annotated TSS at genes of interest were determined via BEDTools (v2.29.2) coverage function using the ‘-counts’ flag, then normalized by reads overlapping ± 1 kb flanks relative to all RefSeq TSS.

### RNA-seq

4-week-old WT mice were fed an ND (n = 4) and an LID (n = 4) for 6 days. Thyroid RNA was extracted using a RNAqueous-Micro total RNA isolation kit (ThermoFisher Scientific). TruSeq Stranded mRNA kit and TruSeq RNA Library preparation kit (Illumina Inc., San Diego, CA) were used to make libraries for RNA-Seq. Sequencing reads were obtained using a NextSeq500 or a NovaSeq 6000 Sequencing System (Illumina). Differential gene expression analysis was carried out through edgeR package. Genes with a minimum of 1.5-fold expression difference and FDR of less than 0.05 were considered as differentially expressed.

### Pathway analysis

Pathway analysis was performed via DAVID Bioinformatics Resources 6.8 (http://david.abcc.nbcifcrf.gog/), ToppGene (http://toppgene.cchmc.org) for KEGG, Reactome, and Biocarta pathway analyses.

### ChIP Q-PCR analysis

ChIP Q-PCR was performed with thyroid glands isolated from WT and *Glis3*KO mice fed a ND using NKX2.1 and PAX8 antibodies. Q-PCR reactions were carried out in triplicate on three independent samples using StepOnePlus Real-time PCR system (Applied Biosystem). *Gapdh* and *Tpo* (at − 1.9 kb) were used as negative controls. Primer sequences are listed in Additional file 5: Table S1.

### Data availability

The PAX8 and NKX2.1 ChIP-seq data from the thyroid glands and the GLIS3-HA, NKX2.1, and FOXE1 ChIP-seq data from PCCl3 cells generated in this study were deposited in the NCBI Gene Expression Omnibus (GEO) database under accession #GSE20777. RNA-seq data from WT mice fed an ND and an LID were deposited under accession #GSE20775. GLIS3 ChIP-Seq data from mouse thyroid glands and RNA-Seq data of thyroid glands from WT and *Glis3*KO mice fed an LID used in this study were from GSE103297. The PAX8 ChIP-Seq data from PCCl3 cells were obtained from GSE26938.

### Statistical analysis

Data are presented as mean ± standard deviation (SD) and were analyzed by one-way ANOVA.

## Results

### TF binding motifs near GLIS3 binding loci

**Fig. 1:**
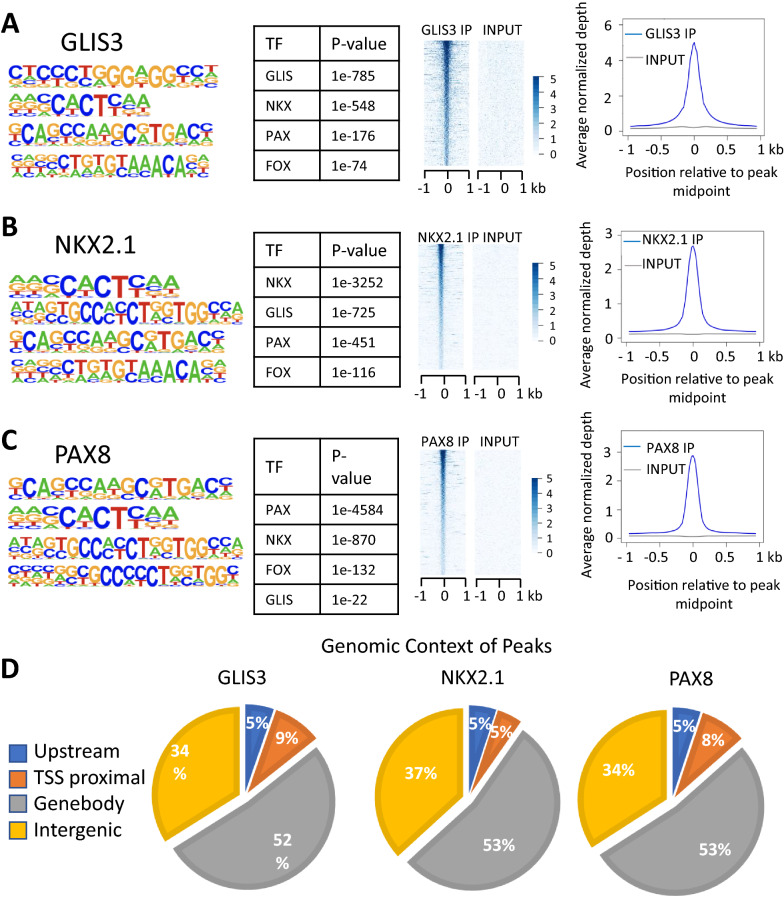
Global analysis of GLIS3, NKX2.1, and PAX8 genomic binding in mouse thyroid glands. **A**–**C** HOMER analysis, heatmap, and ChIP-Seq read density of GLIS3 (**A**), NKX2.1 (**B**), and PAX8 (**C**) binding data. Heatmap of the 2 kb region is centered on each of the binding peaks identified. **D** Genomic context of the GLIS3, NKX2.1, and PAX8 peaks within the whole mouse genome (mm10)

Comprehensive analysis of our GLIS3 ChIP-Seq data of the mouse thyroid gland [19] by HOMER indicated that binding motifs of the NKX, FOX, and PAX TF family are frequently localized near GLIS binding sites (GLISBS) (Fig. 1A). Since NKX2.1, FOXE1, and PAX8 have well-established roles in the regulation of gene expression in thyroid follicular cells [12, 13, 42, 43], we hypothesized that GLIS3 regulates transcription of a subset of target genes in these cells in coordination with these TFs.

To obtain support for this hypothesis, we performed NKX2.1 and PAX8 ChIP-Seq analyses with thyroid glands from mice fed a low iodine diet (LID), in which TSH blood levels are highly elevated [44]. As far as we know, this is the first time ChIP-Seq analyses with thyroid glands and endogenous PAX8 and NKX2.1 have been performed. These analyses identified 29,464 NKX2.1 and 41,044 PAX8 binding peaks. De novo motif analysis of the NKX2.1-enriched sequences identified, in addition to the NKX binding motif, consensus binding sites for members GLIS, PAX, and FOX families were among the top motifs (Fig. 1B). Motif analysis of PAX8 ChIP-Seq data identified a PAX binding sequence as the top binding motif together with binding motifs for NKX, GLIS, and FOX family members (Fig. 1C). NKX2.1 and PAX8 binding loci were most highly enriched within the gene body and intergenic regions, while 5–8% were localized within 1 kb upstream of TSS (TSS proximal) (Fig. 1D).

**Fig. 2:**
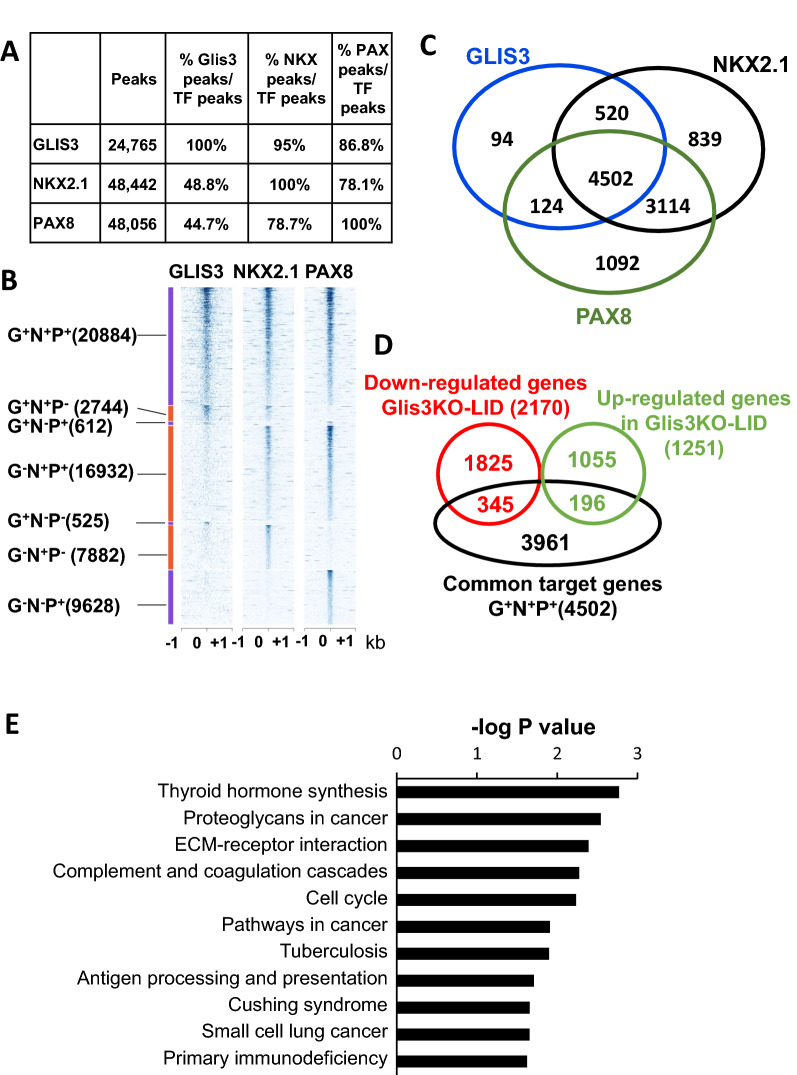
GLIS3, NKX2.1 and PAX8 binding to the mouse thyroid gland genome partially overlap. **A** The number of collapsed binding regions that are positive for GLIS3, NKX2.1, and PAX8 signal, and the percent overlap between them are indicated. **B** Heatmap showing overlap between GLIS3, NKX2.1, and PAX8 binding. Heatmap of the 2 kb region centered on each of the binding regions with ChIP-seq signal normalized to 10 million reads. **C** Venn diagram showing the number of target genes with GLIS3, NKX2.1, and/or PAX8 binding regions. **D** Venn diagram showing the overlap of G^+^N^+^P^+^ genes with genes up- or down-regulated in the thyroid gland of *Glis3*KO-LID mice compared to that of WT-LID mice. **E** KEGG analysis of the 4502 G^+^N^+^P^+^ genes

Comparison of GLIS3, PAX8 and NKX2.1 binding revealed substantial overlaps between GLIS3 binding loci and those of NKX2.1 and PAX8 (Fig. 2A, B). The majority of GLIS3 binding loci contained both NKX2.1 and PAX8 binding regions (referred to as G^+^N^+^P^+^). Subsets of GLIS3 binding regions overlapped with those of either NKX2.1 or PAX8 (G^+^N^+^P^−^ or G^+^N^−^P^+^, respectively), while some of the GLIS3, NKX2.1 and PAX8 binding regions did not exhibit any overlap (G^+^N^−^P^−^, G^−^N^+^P^−^, and G^−^N^−^P^+^, respectively). These observations are consistent with the concept that the transcription of subsets of GLIS3 target genes are regulated in coordination with PAX8 and NKX2.1, and that some genes are regulated by only one or two of the three TFs.

Although enhancers can reside within the gene body and very distant from TSSs, we limited our analysis to genes with binding peaks within 5 kb up- or downstream from the TSS. This analysis identified binding of GLIS3, NKX2.1 and PAX8 near 5240, 8975 and 8832 genes, respectively (Fig. 2C). The majority (85.9%) of the GLIS3-bound genes shared binding both with NKX2.1 and PAX8 (G^+^N^+^P^+^), 9.9% with NKX2.1 only (G^+^N^+^P^−^) and 2.4% with PAX8 only (G^+^N^−^P^+^), while 1.8% bound GLIS3 only (G^+^N^−^P^−^) (Fig. 2C). A summary of GLIS3, NKX2.1, and PAX8 bound genes is presented in Additional file 6: Table S2A.

### GLIS3, NKX2.1, and PAX8 binding to TH biosynthetic and TSH-induced genes

We previously reported that loss of GLIS3 function particularly suppresses the expression of a subset of genes that are required for TH biosynthesis, induced when mice are fed an LID or known to be induced by TSH [11, 19, 45, 46]. Therefore, we were interested in determining which of these differentially expressed genes were regulated by GLIS3 in coordination with PAX8 and/or NKX2.1. Among the 4502 G^+^N^+^P^+^ genes (Fig. 2C, D), 345 and 196 genes were, respectively, down- or up-regulated in *Glis3*KO mice fed an LID (*Glis3*KO-LID) compared to WT mice fed an LID (WT-LID) thyroid (Additional file 6: Table S2B, C). KEGG pathway analysis of the 345 down-regulated G^+^N^+^P^+^ genes identified TH biosynthesis as the top pathway (Fig. 2E). No pathway was found to be significantly associated with G^+^N^+^P^+^ up-regulated genes. Table 1 shows GLIS3, PAX8, and NKX2.1 binding to several gene clusters in relation to their repression in *Glis3*KO-LID thyroid and their induction in WT-LID thyroid. This comparison shows that GLIS3, PAX8, and NKX2.1 bound to many genes critical for TH biosynthesis, including *Slc5a5*, *Slc26a4*, *Tpo*, *Tg*, *Iyd*, and *Duoxa2,* and several genes known to be induced by TSH, such as *Adm2*, *Sod3*, *Dio1*, and *Cdh16* [45–49]. The expression of several of these genes (e.g., *Slc5a5*, *Slc26a4*, *Adm2*, *Sod3*, *Dio1*, *Cdh16*) was significantly repressed in *Glis3*KO-LID mice compared to WT-LID and induced in the thyroid of WT-LID mice compared to WT-ND (Table 1). Other thyroid genes, including *Tpo*, *Iyd*, and *Duoxa2*, were induced in WT-LID thyroid, but not significantly affected by the loss of GLIS3 function, while the expression of certain GLIS3, PAX8, and NKX2.1 bound genes, including *Tg*, *Txnrd1*, and *Duox2*, were not significantly altered in WT-LID thyroid nor suppressed in *Glis3*KO-LID (Table 1).

**Table 1:** GLIS3, NKX2.2, and PAX8 binding to nearby genes (within 5 kb regions from TSS) in comparison to their in/decreased expression in *Glis3*KO-LID or WT-LID thyroid

Category	geneSYM	RNA-Seq FC: LID-WT vs ND-WT	P value	RNA-Seq FC: *Glis3*KO-LID vs WT-LID	P value	GLIS3 target	NKX2.1 target	PAX8 target
TH synthesis	Slc5a5	12.50	3.01E−60	− 15.73	3.81E−28	+	+	+
	Slc26a4	6.45	1.99E−29	− 21.32	1.79E−14	+	+	+
	Tpo	6.08	2.25E−64	NS		+	+	+
	Tshr	2.74	1.36E−08	NS		−	+	+
	Iyd	1.75	5.79E−09	NS		+	+	+
	Slc16a2	− 1.68	2.69E−05	− 2.49	2.84E−33	+	+	−
	Duox1	− 2.39	9.95E−04	NS		−	+	+
	Duoxa1	− 2.84	7.79E−07	NS		−	+	+
	Duox2	NS		NS		+	+	+
	Tg	NS		NS		+	+	+
	Duoxa2	1.72	2.47E−05	NS		+	+	+
TSH induced	Adm2	155.33	1.17E−140	− 271.48	1.03E−267	+	+	+
	Sod3	11.30	4.24E−90	− 18.80	5.28E−113	+	+	+
	Cdh13	9.32	1.10E−138	− 10.59	2.90E−95	−	−	−
	Dio1	3.26	4.44E−49	− 2.32	7.68E−10	+	+	+
	Cdh16	2.57	5.35E−15	− 8.17	4.03E−68	+	+	+
	Pde4d	2.53	5.40E−09	NS		−	−	−
	Ano1	2.49	2.84E−06	NS		−	−	−
	Kcnq1	2.07	8.66E−07	− 2.24	6.78E−13	−	+	+
Thyroid function	Txnrd1	1.88	1.74E−12	NS		+	+	+
	Txnrd2	− 1.58	3.07E−05	NS		+	+	+
	Gnas	− 1.61	1.04E−06	NS		+	+	+
	Fam20c	− 2.30	1.92E−05	NS		+	+	+
	Clcn5	NS		NS		−	−	−
	Dio2	NS		NS		−	−	−
	Gnaq	NS		NS		+	+	+
	Gpx1	NS		NS		+	+	+
	Kcne2	NS		NS		−	−	−
	Txn1	NS		NS		+	+	+
	Txn2	NS		NS		−	−	−
Thyroid TFs	Glis3	2.10	2.22E−07	NS		+	+	+
	Hhex	NS		NS		+	+	+
	Pax8	NS		2.03	2.01E−15	+	+	+
	Nkx2-1	NS		NS		+	+	+
	Foxe1	NS		NS		+	+	+
ECM	Adamts8	22.02	3.27E−03	− 57.44	2.54E−84	−	−	−
	Itga2	14.29	5.60E−68	− 47.14	4.50E-58	−	−	−
	Col4a1	4.43	1.24E−34	− 8.55	1.37E−91	−	+	+
	Cdh5	3.58	9.83E−30	− 3.98	5.85E−47	−	−	−
	Col18a1	3.48	2.55E−19	− 8.22	1.05E−105	+	+	+
	Col1a1	3.36	7.67E−15	− 37.69	2.92E−37	−	−	−
	Col4a2	3.36	1.86E−25	− 4.80	1.70E−44	−	+	+
	Col13a1	3.18	1.95E−46	NS		−	+	−
	Col5a2	3.13	1.42E−21	− 12.68	1.55E−78	−	−	−
	Col1a2	2.85	6.95E−14	− 17.43	2.35E−46	−	−	−
	Col3a1	2.76	2.04E−13	− 37.92	1.10E−53	−	−	−
	Col5a3	2.67	9.43E−11	− 5.56	5.77E−53	+	+	+
	Col11a2	2.49	8.43E−17	− 2.27	3.60E−06	−	−	−
	Col6a1	2.38	4.39E−11	− 3.78	3.77E−06	−	−	−
	Col16a1	2.17	1.84E−14	− 5.91	1.75E−60	−	+	+
	Col6a2	2.15	1.47E−07	NS		−	+	+
	Col14a1	2.02	8.30E−10	− 10.66	4.56E−34	−	−	−
	Col12a1	1.90	3.66E−06	NS		+	+	+
	Col15a1	1.44	4.16E−03	− 4.68	1.73E−47	+	+	+
	Itgb4	NS		NS		+	+	+
	Cdh4	NS		− 2.34	1.47E−18	+	+	+
Inflammation	Ccl2	21.36	4.44E−49	− 37.84	7.27E−25	−	−	−
	Ccl7	12.97	2.13E−46	− 85.29	7.65E−47	−	−	−
	Ccl17	3.64	7.08E−04	− 29.32	1.01E−14	−	−	−
	Ccl6	3.05	2.62E−11	− 3.23	2.15E−17	−	−	−
	Ccl9	2.69	1.15E−10	− 3.20	4.10E−15	+	+	+
	Ccl8	2.39	6.85E−04	− 4.47	2.19E−06	−	−	−
	Ccl12	2.22	1.10E−03	− 30.15	1.65E−18	−	−	−
	Il6	5.62	7.32E−07	− 14.73	2.75E−10	−	−	−

+: indicates binding of respective TF; −: indicates no binding

*NS* no significant change

**Fig. 3:**
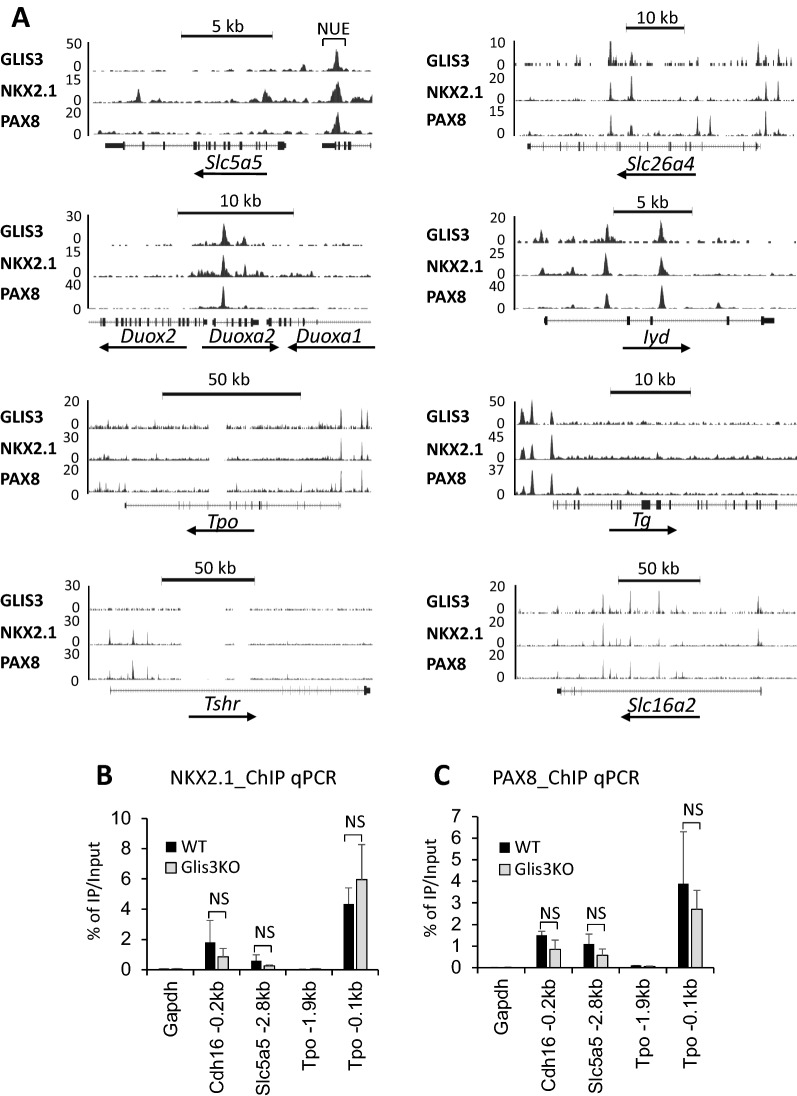
GLIS3, NKX2.1, and PAX8 share binding loci within the regulatory regions of several TH biosynthetic genes. **A** Colocalization of GLIS3, NKX2.1, and/or PAX8 ChIP-seq loci in genes critical for TH biosynthesis. The NUE region in *Slc5a5* (*Nis*) is indicated. **B**, **C** NKX2.1 and PAX8 analysis at *Cdh16* (− 0.2 kb), *Slc5a5* (− 2.8 kb), and *Tpo* (− 0.1 kb) with thyroid glands from WT and *Glis3*KO mice. Binding to *Gapdh* and *Tpo* (− 1.9 kb) served as negative controls

The genome browser tracks in Fig. 3A indicate the shared locations of the binding of endogenous PAX8, NKX2.1, and GLIS3 in several genes critical for TH biosynthesis, including *Slc5a5*, *Slc26a4*, *Duoxa2*, *Iyd*, *Tpo*, and *Tg*, and *Slc16a2*. In several genes (e.g., *Slc5a5*, *Slc26a4*, *Tpo*, *Tg*), PAX8, NKX2.1, and GLIS3 bound within the same region of the proximal promoter. In *Slc16a2* and *Iyd* only NKX2.1 and GLIS3 shared binding to the proximal promoter region, while NKX2.1 and PAX8 bound to *Tshr*, but not GLIS3. In several genes (e.g., *Tpo*, *Tg*, *Slc26a4*, *Iyd*, *Slc16a2*) binding of these 3 TFs overlapped in more than one region suggesting that their transcription may be controlled by multiple regulatory regions (Fig. 3A). These observations support the hypothesis that GLIS3 regulates gene transcription in coordination with several other thyroid TFs. In the case of *Duoxa2*, the GLIS3/PAX8/NKX2.1 binding region is in intron 1 of *Duoxa2*, which is within a 35 kb region on mouse chromosome 2 that also encompasses *Duox2* and *Duoxa1* (Fig. 3A)*.* We cannot rule out that his enhancer region might play a role in the transcriptional regulation of all three genes.

The transcriptional regulation of *Slc5a5* has been extensively studied in thyroid follicular cell lines and reported to be controlled by the proximal promoter and a region, referred to as *Nis* upstream enhancer (NUE, located − 2.8 kb from the TSS) that has been reported to bind several TFs, including PAX8 and NKX2.1 [13, 14, 43, 50–55]. The *Slc5a5* genome browser tracks show the localization of binding peaks for endogenous GLIS3, PAX8, and NKX2.1 within the NUE region, whereas no major binding was observed within the proximal promoter region (Fig. 3A). These observations are consistent with the view that NUE is a major enhancer region driving the activation *Slc5a5* transcription by GLIS3 in the thyroid of mice fed an LID*.* As *Scl5a5* is one of the genes most strongly regulated by GLIS3 (Table 1), this raised the question whether GLIS3 binding was required for the binding of PAX8 and NKX2.1 to the NUE region. To investigate this, we compared NKX2.1 and PAX8 binding to the NUE region in thyroids isolated from WT and *Glis3*KO mice. ChIP Q-PCR analysis demonstrated that NKX2.1 and PAX8 occupancy at the NUE region was not significantly affected by the absence of GLIS3 (Fig. 3B, C) indicating that GLIS3 is not required for PAX8 or NKX2.1 binding to the NUE regulatory region. The lack of GLIS3 also did not significantly affect PAX8 or NKX2.1 binding to the proximal promoters of *Cdh16* and *Tpo* (region − 0.1 kb upstream from TSS). No significant binding of PAX8 or NKX2.1 was observed to *Gapdh* or the − 1.9 kb upstream region of *Tpo*, which served as negative controls (Fig. 3B, C). ChIP-Seq analysis of several epigenetic markers showed no significant differences in the level of H3K4me3 and H3K27me3 signals in GLIS3-bound genes that were differentially expressed between Glis3KO-LID and WT-LID thyroid glands (Additional file 1: Fig. S1A). Moreover, little difference in H3K4me3 and H3K27me3 signals was observed at the NUE region of *Slc5a5* between WT and *Glis3*KO thyroid glands, although some small changes in these signals were observed at its proximal promoter (Additional file 1: Fig. S1B). Together, these observations suggest that in the absence of GLIS3 genomic regions, such as NUE, remain accessible and in an active (open) state.

**Fig. 4:**
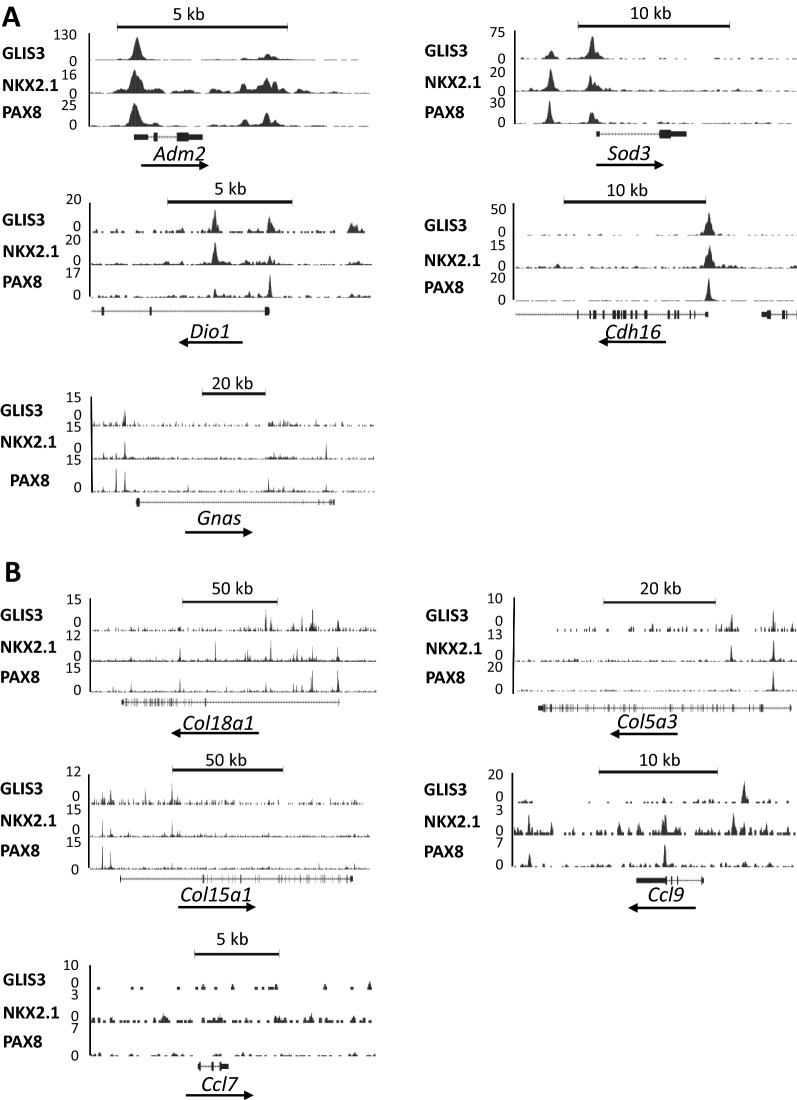
Genome browser tracks of several genes showing overlap of GLIS3, NKX2.1, and PAX8 binding loci in mouse thyroid gland. **A** Genes known to be induced by TSH. **B** Collagen and chemokine genes

Genome browser tracks in Fig. 4A show the overlap between the binding of endogenous PAX8, NKX2.1, and GLIS3 to regulatory regions of several other thyroid genes reported to be induced by TSH, induced in WT-LID, and repressed in *Glis3*KO-LID thyroids, including *Adm2*, *Sod3*, *Dio1*, and *Cdh16* [45–48].

GLIS3, NKX2.1, and PAX8 binding to extracellular matrix (ECM) and inflammatory genes.

Next, we examined whether any differentially expressed ECM and inflammatory genes are co-regulated by GLIS3, PAX8, and/or NKX2.1. In contrast to TH biosynthetic genes, relatively few ECM and inflammatory genes (e.g., *Col18a1*, *Ccl9*) showed binding of all 3 TFs, while a few genes (e.g., *Col4a1*, *Col16a1*) bound PAX8 and NKX2.1, but not GLIS3 (Table 1). Of course, we cannot rule out that these TFs might regulate some of these genes by binding distant enhancers. The genome browser tracks in Fig. 4B show overlaps between GLIS3, PAX8, and NKX2.1 binding regions in *Col18a1*, *Col5a3*, *Col15a1*, *Col4a2*, and *Ccl9*. The transcription of other differentially expressed ECM and inflammatory genes, such as *Ccl7*, that do not show GLIS3, PAX8, and NKX2.1 binding, are likely regulated by other TFs.

Our ChIP-Seq analysis further identified binding peaks of all three TFs within the same genomic region(s) of *Glis3*, *Pax8*, and *Nkx2.1*, as well as two other thyroid TF genes, *FoxE1* and *Hhex* (Additional file 2: Fig. S2) suggesting transcriptional regulation of each other consistent with previous observations [3, 4, 56].

### GLIS3 regulates a subset of TH biosynthetic genes in coordination with FOXE1

**Fig. 5:**
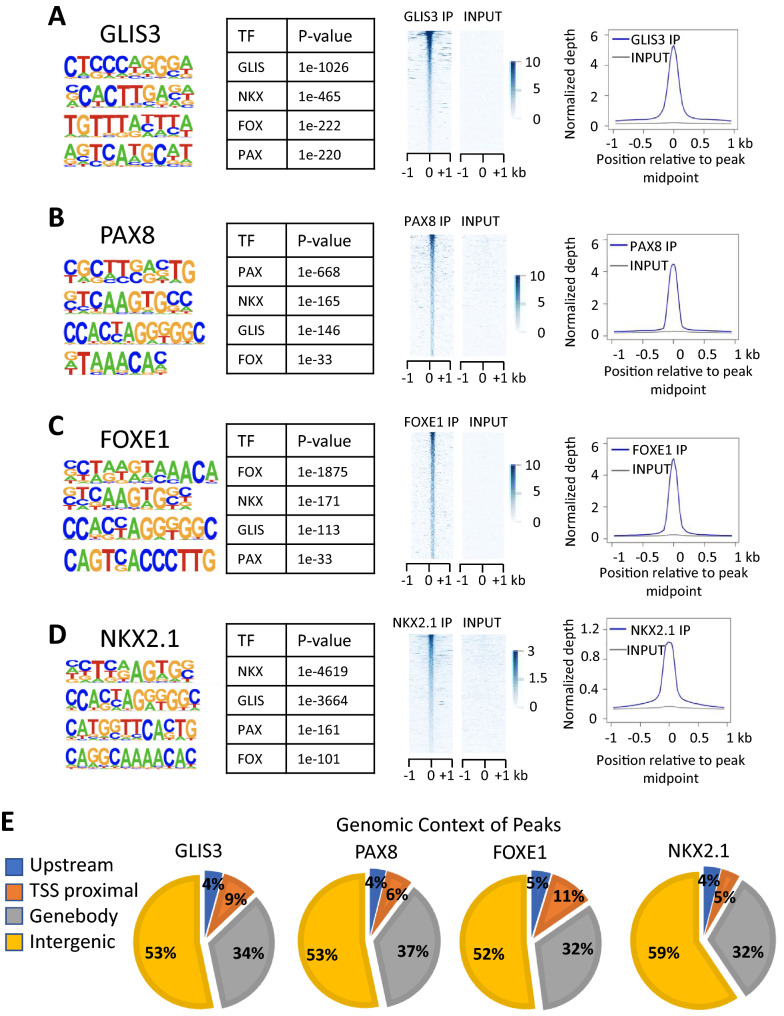
Global analysis of GLIS3, NKX2.1, PAX8, and FOXE1 genomic binding in rat thyroid follicular PCCl3 cells. **A**–**D** HOMER analysis, heatmap, and ChIP-Seq read density of GLIS3 (**A**), PAX8 (**B**), FOXE1 (**C**), and (**D**) NKX2.1 binding data. **E** Genomic context of the GLIS3, PAX8, FOXE1, and NKX2.1 peaks within the whole rat genome (rn6)

**Fig. 6:**
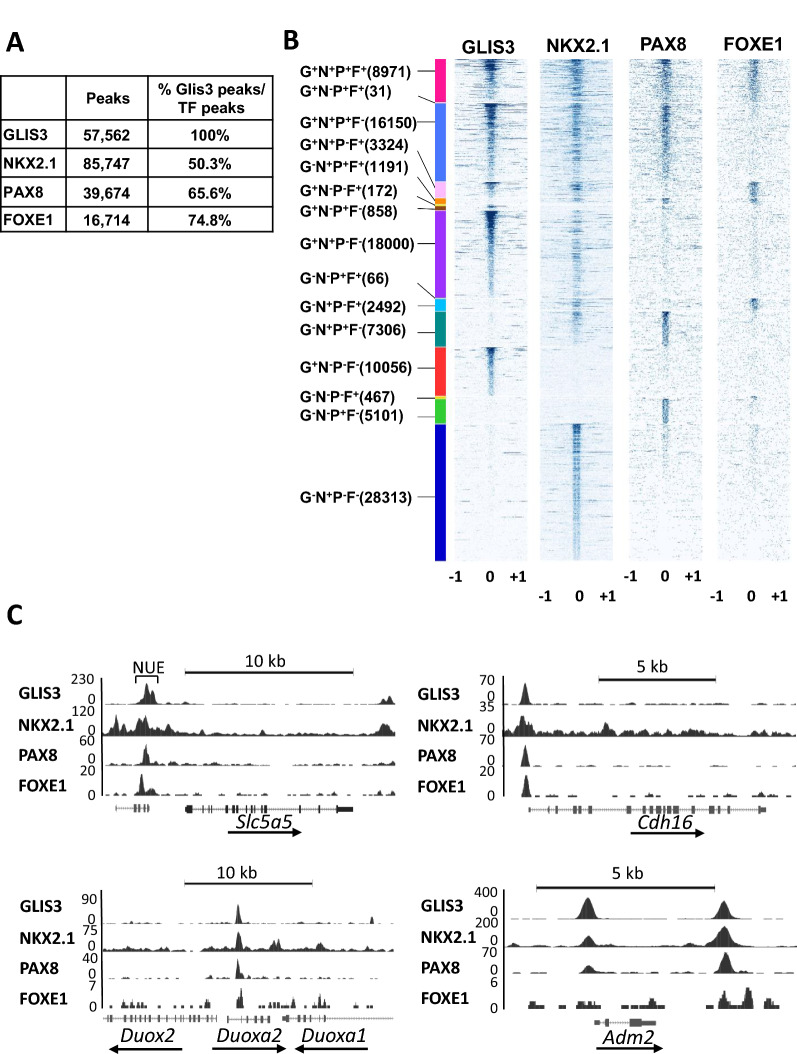
GLIS3, NKX2.1, PAX8, and FOXE1 binding to the PCCl3 genome partially overlap. **A** Percent NKX2.1, PAX8, and FOXE1 binding loci overlapping with those of GLIS3. **B** Heatmaps of the 2 kb region centered on each of the binding regions with ChIP-seq signal normalized to 10 million reads shows overlaps between GLIS3, NKX2.1, PAX8, and FOXE1 binding. **C** Genome browser tracks of several thyroid genes showing overlap of GLIS3, NKX2.1, PAX8, and FOXE1 binding loci in PCCl3 cells

Since HOMER also identified FOX binding motifs near GLISBS (Fig. 1A), we were interested in examining co-regulation of TH biosynthetic genes by GLIS3 and FOXE1. Since several attempts to perform FOXE1 ChIP-Seq analysis in mouse thyroid glands were unsuccessful, we performed FOXE1, NKX2.1, and GLIS3-HA ChIP-Seq analysis in rat thyrocyte PCCl3 cells and compared these data with that of PAX8 in PCCl3 cells reported previously [43]. Nuclear expression of GLIS3-HA and endogenous FOXE1 in PCCl3 was confirmed by immunofluorescence staining (Additional file 3: Fig. S3). Consistent with our de novo motif analysis of the GLIS3 ChIP-Seq data from the mouse thyroid gland, analysis of the GLIS3-enriched sequences identified GLISBS as the top motif as well as consensus binding motifs for NKX, FOX, and PAX family members (Fig. 5A). Similarly, de novo motif analysis of the NKX2.1, PAX8 and FOXE1-enriched sequences identified, in addition to their own consensus binding motif, binding motifs of the three other TFs (Fig. 5B–D). The genomic contexts of the GLIS3, FOXE1, PAX8, and NKX2.1 binding peaks are shown in Fig. 5E. Comparison of GLIS3, PAX8, NKX2.1, and FOXE1 binding revealed substantial overlaps between the GLIS3 binding loci and the other three TFs (Fig. 6A, B). Most FOXE1-binding loci (74.8%) were in the proximity of GLIS3-binding loci. The different clusters of GLIS3, NKX2.1, PAX8 and/or FOXE1 bound genes (based on regions within 5 kb from the TSS) are presented in Additional file 7: Table S3. The data showed that GLIS3, NKX2.1, PAX8, and FOXE1 (G^+^N^+^P^+^F^+^) were bound near each other in 3118 genes, including several TH biosynthetic and TSH-responsive genes [e.g., *Scl5a5* (NUE region), *Duoxa2*, *Duox2*, *Cdh16,* as well as *Pax8*, *Nkx2.1*, *Hhex*, and *Foxe1*], but not several other G^+^N^+^P^+^ thyroid genes (e.g., *Slc16a2*, *Tshr*, and *Dio1*). The genome browser tracks display the co-localization of GLIS3, NKX2.1, PAX8, and FOXE1 binding peaks in *Scl5a5*, *Cdh16*, *Duoxa2*, and *Adm2* (Fig. 6C) and *Glis3*, *Nkx2.1*, *Pax8*, and *Foxe1* (Additional file 4: Fig. S4) in PCCl3 cells. FOXE1 binding did not overlap with GLIS3, PAX8, and NKX2.1 binding at the proximal promoter of *Adm2* but did show an overlap within a downstream intergenic region. The GLIS3, NKX2.1, and PAX8 binding patterns in PCCl3 were very similar to those as observed in mouse thyroid (Fig. 3A; Additional file 2: Fig. S2). However, binding of GLIS3, NKX2.1, and PAX8 in PCCl3 cells did not always match those observed in the mouse thyroid gland, including their binding to *Tpo*, *Glis3*, and *Iyd* (Table 2). This might be due to epigenomic differences between mouse thyroid follicular cells in vivo and immortalized rat thyrocyte PCCl3 cells, for which the gene expression profile is likely different from thyroid follicular cells in vivo.

**Table 2:** GLIS3, NKX2.2, PAX8, and FOXE1 to nearby genes (within 5 kb regions of TSS) in rat thyroid follicular PCCl3 cells

Category	geneSYM	rGLIS3 target	rNKX2.1 target	rPAX8 target	rFOXE1 target
TH synthesis	Slc5a5	+	+	+	+
	Slc26a4	−	+	+	+
	Tpo	−	+	−	−
	Tshr	+	+	+	−
	Iyd	−	−	−	−
	Slc16a2	+	+	+	−
	Duox1	−	+	−	−
	Duoxa1	+	+	−	−
	Duox2	+	+	+	+
	Tg	−	+	−	−
	Duoxa2	+	+	+	+
TSH induced	Adm2	+	+	+	−
	Sod3	+	+	+	+
	Cdh13	−	+	−	−
	Dio1	+	+	+	−
	Cdh16	+	+	+	+
	Pde4d	+	+	+	+
	Ano1	+	+	+	−
	Kcnq1	−	−	−	−
Thyroid function	Txnrd1	+	+	+	+
	Txnrd2	+	+	+	−
	Gnas	+	+	+	−
	Fam20c	+	+	+	+
	Clcn5	−	−	−	−
	Dio2	−	−	−	−
	Gnaq	+	+	+	−
	Gpx1	+	+	−	+
	Kcne2	−	−	−	−
	Txn1	+	+	+	−
	Txn2	+	+	+	−
Thyroid TFs	Glis3	−	−	−	−
	Hhex	+	+	+	+
	Pax8	+	+	+	+
	Nkx2-1	+	+	+	+
	Foxe1	+	+	+	+

## Discussion

Comprehensive HOMER analysis of our GLIS3 ChIP-Seq data from the mouse thyroid gland indicated that members of the PAX, NKX, and FOX families frequently bind in the proximity of GLIS3 binding loci. We proposed that GLIS3 regulates the transcription of certain thyroid genes in coordination with PAX8, NKX2.1, and FOXE1, which have well-established regulatory functions in the thyroid, including thyroid gland development and TH biosynthesis [4, 7, 10, 12, 13, 30]. And although binding of PAX8, NKX2.1, and FOXE1 to a few thyroid genes, such as *Slc5a5*, has been demonstrated by in silico and ChIP-PCR analyses, global ChIP-Seq analysis of the binding of endogenous PAX8, NKX2.1, FOXE1 has not been previously reported. Our GLIS3, PAX8, NKX2.1, and FOXE1 cistrome analyses with mouse thyroid gland and/or thyrocyte PCCl3 cells identified considerable overlaps in binding of these four TFs indicating that GLIS3 shares many of its promoter/enhancer regions with PAX8, NKX2.1, and/or FOXE1, including genes related to thyroid biosynthesis/function and/or induced by TSH (Figs. 2 and 6). Our data are consistent with previous in silico and ChIP-PCR analyses indicating that binding motifs for these thyroid TFs localize near each other within the regulatory region of certain TH biosynthetic genes [4, 42, 57]. Thyroid target genes are regulated by different combinations of these TFs thereby forming distinct clusters, e.g., G^+^N^+^P^+^F^+^, G^+^N^+^P^+^F^−^, etc. (Additional file 7: Table S3A). Although the expression of some G^+^N^+^P^+^ genes was not or only moderately changed, genes most highly induced in WT-LID thyroid (versus WT-ND) or repressed in *Glis3*KO-LID thyroid (versus WT-LID), including *Slc5a5*, *Slc26a4*, *Adm2*, *Sod3*, and *Cdh16*, all showed shared GLIS3, PAX8, NKX2.1 binding (Table 1; Fig. 3A) and in PCCl3 cells, frequently overlap with FOXE1 binding sites (Table 2; Fig. 6C). Together, these findings support our hypothesis that GLIS3 regulates these genes in coordination with PAX8, NKX2.1, and/or FOXE1 and further demonstrate that GLIS3 binding to these shared regulatory regions is essential for optimal expression of these genes and their induction by TSH. Except for a few genes (e.g., *Col18a1*, *Col5a3*, *Ccl9*) (Table 1; Fig. 4B), GLIS3, PAX8, and NKX2.1 did not bind near many of the differentially expressed ECM and chemokine genes suggesting that they do not play a direct role in the transcriptional regulation of many of these genes. However, we cannot rule out that they might be regulated by distant binding sites as may be the case for *Ccl7*, which has an NKX2.1 binding site 62 kb upstream from its TSS. Interestingly, in thyroid follicular cells colocalization of GLIS3, PAX8, and NKX2.1 binding peaks was also found in *Glis3*, *Pax8* and *Nkx2.1*, as well as in *Foxe1* and *Hhex*, suggesting that these TFs may coregulate each other’s expression. This is consistent with previous studies showing that during thyroid development these TFs are part of an integrated regulatory network in which each of them controls the expression of other members [3, 4, 56]. In mice, NKX2.1 and PAX8 are expressed at an earlier stage of embryonic thyroid development [4, 12] than GLIS3 (manuscript in preparation). This would be consistent with the hypothesis that during embryonic thyroid development *Glis3* transcription is directly regulated by NKX2.1 and PAX8.

An interesting question was whether GLIS3 is required for the binding of PAX8 and NKX2.1 to common regulatory regions and affects the open/closed chromatin structure. Comparison of the binding of PAX8 and NKX2.1 to the NUE region of *Slc5a5* and the proximal promoter of *Cdh16* and *Tpo* in the thyroid gland from WT and *Glis3*KO mice by ChIP-PCR indicated that lack of GLIS3 has no significant effect on the binding of PAX8 and NKX2.1 to the NUE and the two other proximal promoter regions. These data suggest that GLIS3 does not greatly affect the open/closed chromatin structure at these regions. This was supported by our analysis of histone markers, H3K4me3 and H3K27me3, which reflect active and inactive transcription, showing little difference in the intensity of the signals around the NUE region and in the meta-analysis of H3K4me3 and H3K27me3 between WT-LID and *Glis3*KO-LID. However, we cannot rule out that GLIS3 binding alters the chromatin of certain genes. Future studies using scATAC-seq analysis might establish whether GLIS3 has any effect on chromatin structure.

**Fig. 7:**
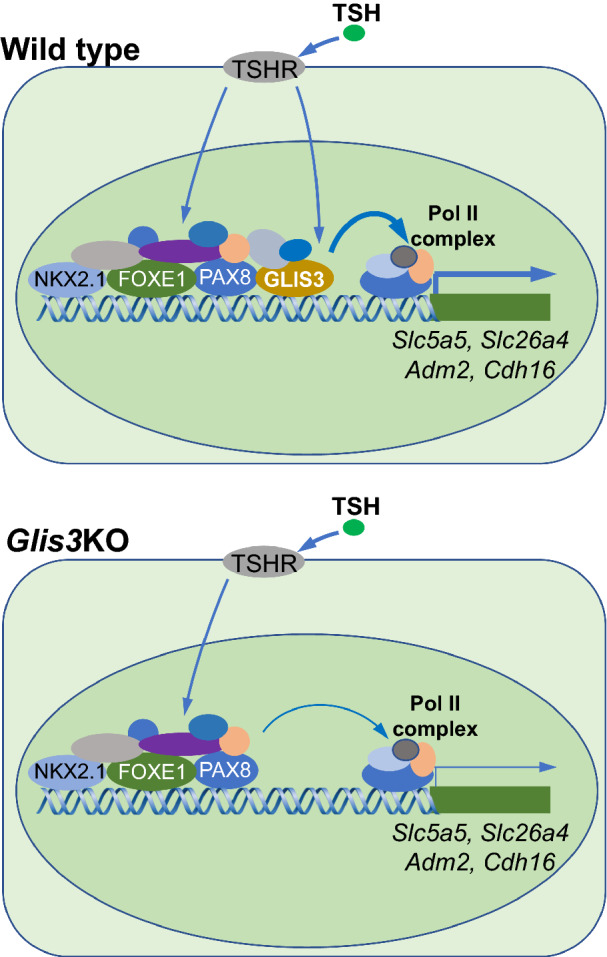
Schematic view of GLIS3 regulation in the expression of target genes with other TFs. GLIS3 does not affect PAX8 or NKX2.1 binding nor the open/closed chromatin structure at their regulatory regions of several GLIS3 target genes but may promote transcriptional activation by enhancing the interaction of regulatory regions with Pol II complexes

GLIS3 is particularly critical for the transcriptional regulation of several genes that are highly induced in WT-LID thyroid and repressed in *Glis3*KO-LID thyroid, including *Slc5a5*, *Slc26a4*, *Cdh16*, *Adm2*, and *Sod3* (Fig. 7). We hypothesize that GLIS3 boosts the transcriptional activation of these genes by recruiting additional transcriptional mediators at these regulatory hubs and promoting the interaction with TFs bound to other regulatory hubs through changes in 3D chromatin organization (genome loop formation) thereby enhancing the interaction with polymerase II complexes (Fig. 7) [58]. The transcriptional mediator WWTR1 (TAZ), which interacts directly with GLIS3 and enhances its transcriptional activity [59], might be one of such proteins. WWTR1, which can bind several other thyroid TFs, including the Hippo pathway TF, TEAD1, might facilitate interactions between the NUE-bound transactivation complex with that associated with the *Slc5a5* proximal promoter [13, 60]. Additional studies are needed to establish the role of GLIS3 in these interactions.

## Conclusions

Our data supports the hypothesis that GLIS3 regulates the transcription of many thyroid genes, particularly TH biosynthesis-related and TSH-inducible genes, in coordination with several other thyroid follicular cell-associated TFs by interacting within the same distinct regulatory regions. Although GLIS3 is critical for the TSH-dependent induction of several TH biosynthetic genes, it does not affect the binding of PAX8 or NKX2.1 nor the open/closed chromatin structure. GLIS3 may induce transcriptional activation by promoting the interaction of regulatory regions with other enhancer sites and/or with RNA Polymerase II (Pol II) complexes.

## Supplementary Information

**Additional file 1: Figure S1.** ChIP-Seq analysis of H3K4me3 and H3K27me3 in thyroid glands from WT and *Glis3*KO mice fed a ND or LID. **A** Tukey box- and whisker plots of H3K4me3 and H3K27me3 ChIP-seq signals associated with GLIS3 target genes that are either down- or up-regulated in *Glis3*KO-LID compared to WT-LID thyroids. **B** Genome browser tracks of H3K4me3 and H3K27me3 histone markers at the *Slc5a5* and *Tg* loci in thyroid glands from WT-ND, WT-LID, *Glis3*KO-ND, and *Glis3*KO-LID mice.

**Additional file 2: Figure S2.** Genome browser tracks of *Glis3*, *Nkx2.1*, *Pax8, Foxe1,* and *Hhex* loci showing colocalization of GLIS3, NKX2.1, and/or PAX8 ChIP-seq signal in mouse thyroid glands.

**Additional file 3: Figure S3.** Nuclear expression of endogenous FOXE1 and exogenous GLIS3-HA in PCCl3 cells. Green, FOXE1 or GLIS3-HA; Blue, Dapi.

**Additional file 4: Figure S4.** Genome browser tracks of *Glis3*, *Nkx2.1*, *Pax8*, and *Foxe1* loci showing colocalization of GLIS3, NKX2.1, PAX8 and/or FOXE1 ChIP-seq signal in rat thyrocyte PCCl3 cells.

**Additional file 5: Table S1.** List of primers used in ChIP Q-PCR.

**Additional file 6: Table S2.** **A** Lists of genes binding GLIS3, NKX2.1, and/or PAX8 as indicated (within 5 kb from TSS). ChIP-Seq analysis was performed with thyroid glands from mouse fed an LID. G^+^N^+^P^+^, G^+^N^+^P^−^, etc. are defined as described in “Results” section. **B** Total list of G^+^N^+^P^+^ genes that are down-regulated in *Glis3*KO-LID thyroid gland compared to WT-LID thyroid. **C** Total list of G^+^N^+^P^+^ genes that are up-regulated in *Glis*3KO-LID thyroid gland compared to WT-LID thyroid.

**Additional file 7: Table S3.** Lists of genes binding GLIS3, NKX2.1, PAX8, and/or FOXE1 as indicated (within 5 kb from TSS). ChIP-Seq analysis was performed with rat thyrocyte PCCl3 cells. G^+^N^+^P^+^, G^+^N^+^P^−^, etc. are defined as described in “Results” section. PAX8 data were obtained from [43].

## Data Availability

The datasets used and/or analysed during the current study are available from the corresponding author on reasonable request. Data have been deposited in the NCBI Gene Expression Omnibus (GEO) database under accession numbers GSE20777, GSE20775, GSE103297and GSE26938.

## Abbreviations

GLIS3: Gli-similar 3
GLISBS: GLIS binding sites
CH: Congenital hypothyroidism
TH: Thyroid hormone
TF: Transcriptional factors
TSH: Thyroid stimulating hormone
TSHR: TSH receptor
NIS: Sodium–iodide symporter
NUE: *Nis* upstream enhancer
DUOX2: Dual oxidase 2
TG: Thyroglobulin
TPO: Thyroid peroxidase
PDS: Pendrin
PAX8: Paired box 8
NKX2.1: NK2 homeobox 1
FOXE1: Forkhead box E1
HHEX: Hematopoietically expressed homeobox
HOMER: Hypergeometric optimization of motif enrichment
LID: Low iodine diet
ND: Normal diet
ChIP: Chromatin immunoprecipitation
