# Supplementary figures and images for: GLIS3 regulates transcription of thyroid hormone biosynthetic genes in coordination with other thyroid transcription factors
Source: Cell Biosci. 2023 Feb 15;13:32. doi: 10.1186/s13578-023-00979-8 (PMC9930322; doi:10.1186/s13578-023-00979-8)

A

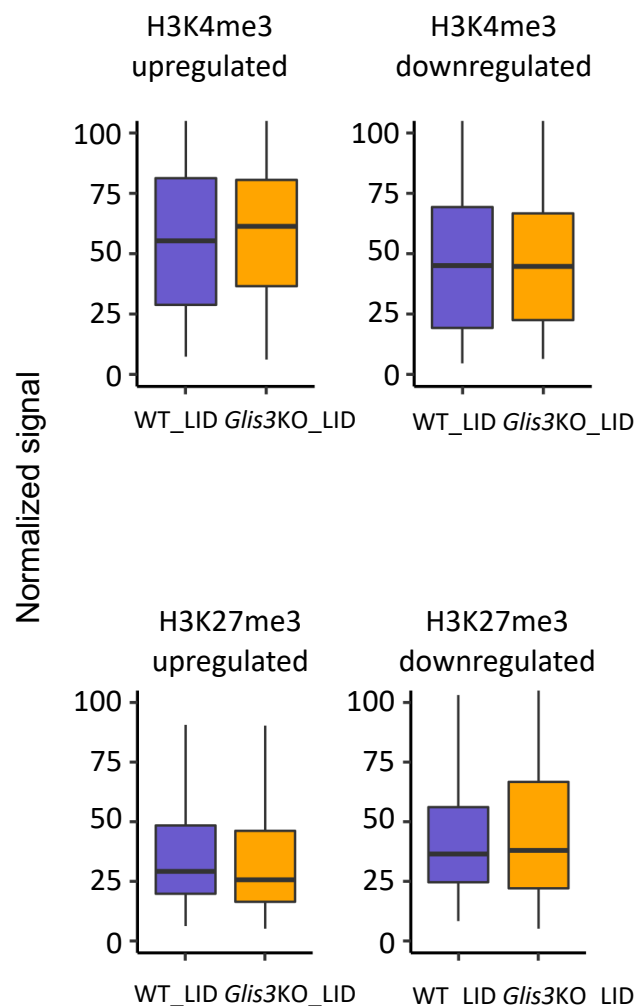

B

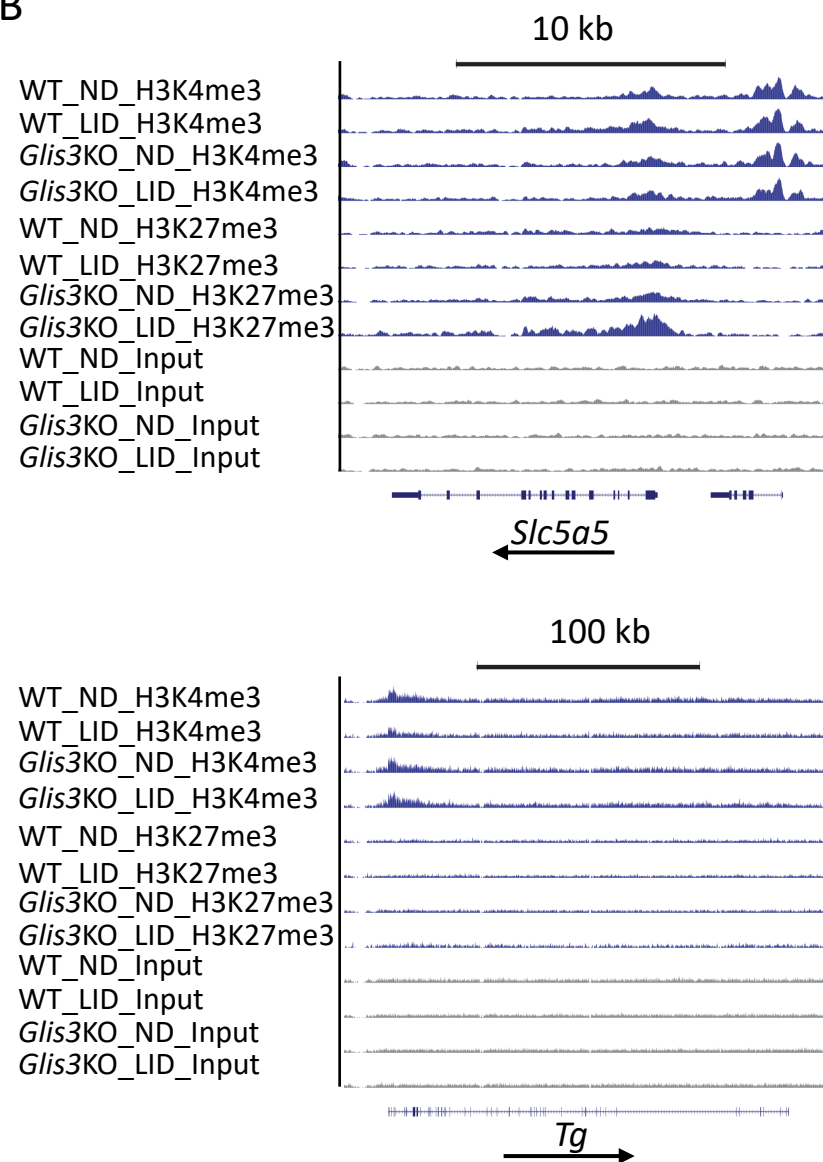

Fig. S1

Supplement: Supplementary file 1 — Additional file 1: Figure S1. ChIP-Seq analysis of H3K4me3 and H3K27me3 in thyroid glands from WT and Glis3KO mice fed a ND or LID. A Tukey box- and whisker plots of H3K4me3 and H3K27me3 ChIP-seq signals associated with GLIS3 target genes that are either down- or up-regulated in Glis3KO-LID compared to WT-LID thyroids. B Genome browser tracks of H3K4me3 and H3K27me3 histone markers at the Slc5a5 and Tg loci in thyroid glands from WT-ND, WT-LID, Glis3KO-ND, and Glis3KO-LID mice. [file 13578_2023_979_MOESM1_ESM.pdf]

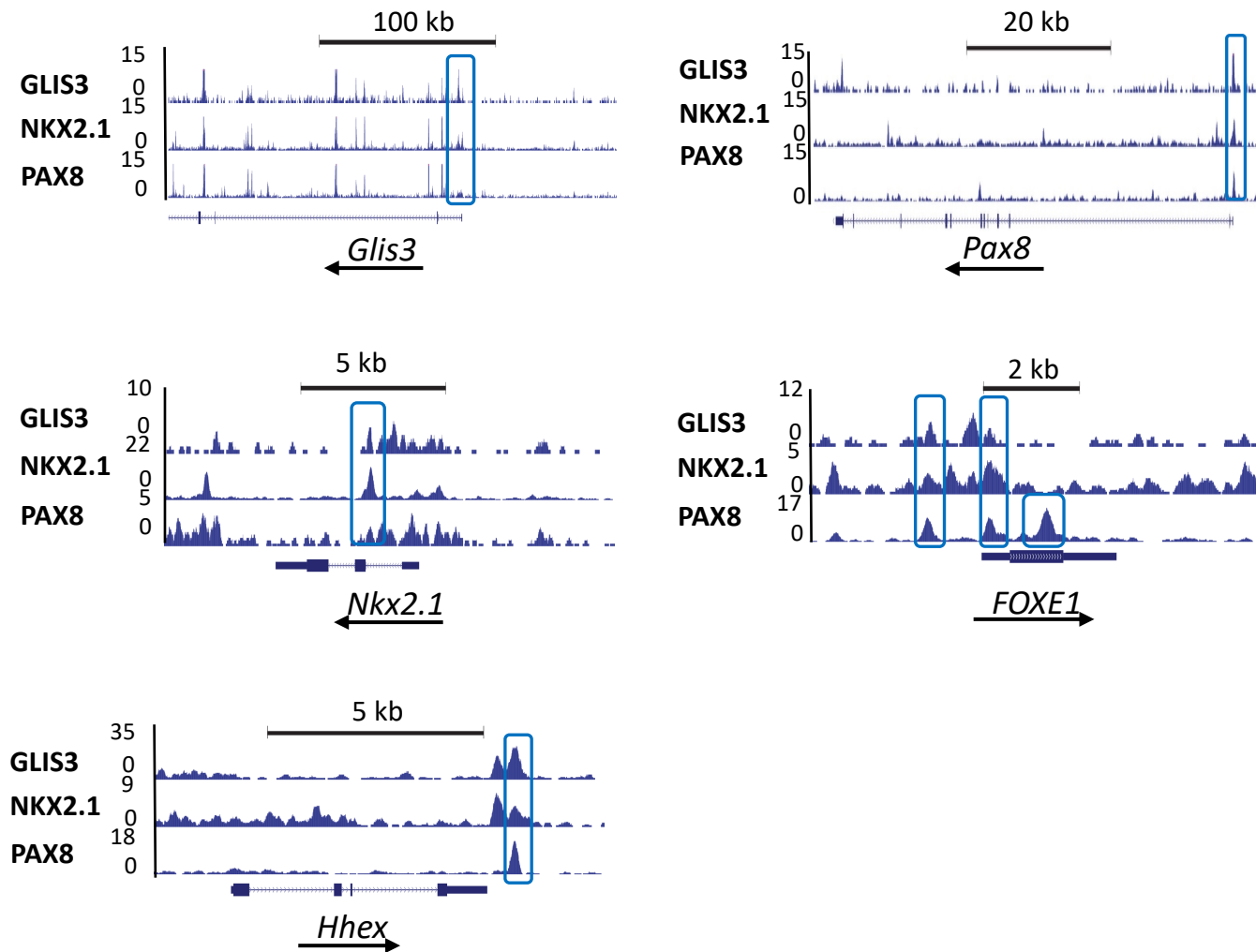

Fig. S2

Supplement: Supplementary file 2 — Additional file 2: Figure S2. Genome browser tracks of Glis3, Nkx2.1, Pax8, Foxe1, and Hhex loci showing colocalization of GLIS3, NKX2.1, and/or PAX8 ChIP-seq signal in mouse thyroid glands. [file 13578_2023_979_MOESM2_ESM.pdf]

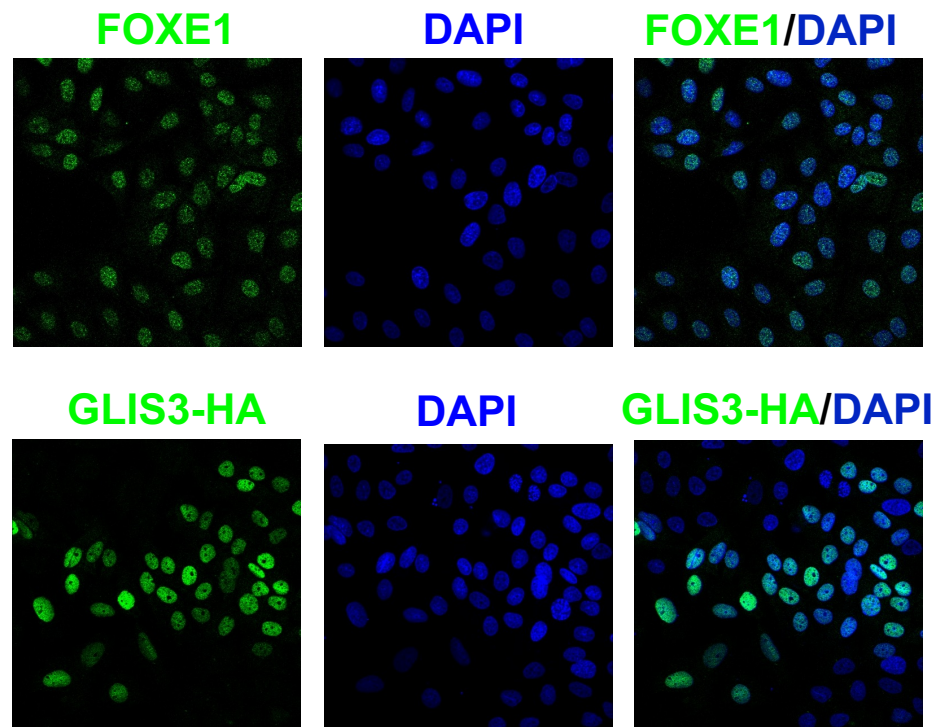

Fig. S3

Supplement: Supplementary file 3 — Additional file 3: Figure S3. Nuclear expression of endogenous FOXE1 and exogenous GLIS3-HA in PCCl3 cells. Green, FOXE1 or GLIS3-HA; Blue, Dapi. [file 13578_2023_979_MOESM3_ESM.pdf]

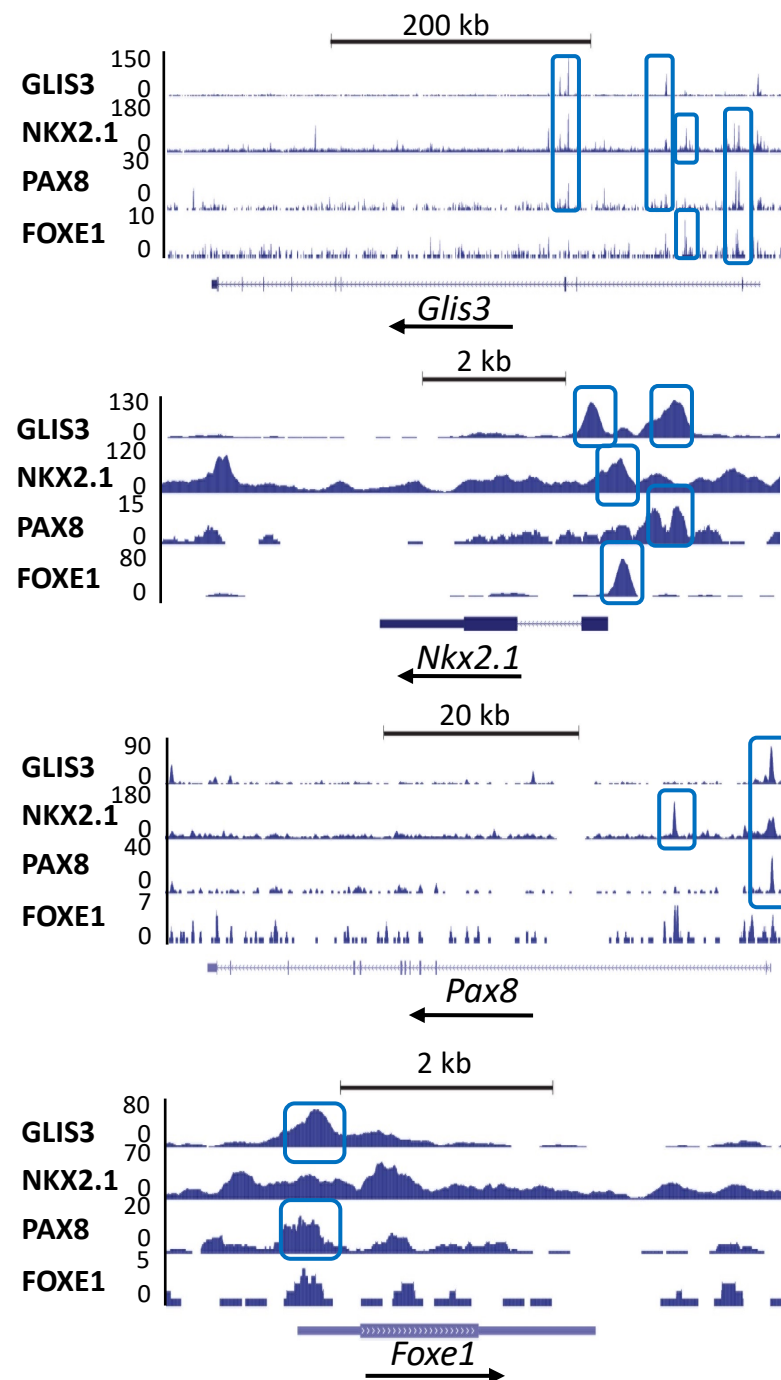

Fig. S4

Supplement: Supplementary file 4 — Additional file 4: Figure S4. Genome browser tracks of Glis3, Nkx2.1, Pax8, and Foxe1 loci showing colocalization of GLIS3, NKX2.1, PAX8 and/or FOXE1 ChIP-seq signal in rat thyrocyte PCCl3 cells. [file 13578_2023_979_MOESM4_ESM.pdf]
